# Agronomic Biofortification of Cayenne Pepper Cultivars with Plant Growth-Promoting Rhizobacteria and Chili Residue in a Chinese Solar Greenhouse

**DOI:** 10.3390/microorganisms9112398

**Published:** 2021-11-21

**Authors:** Ibraheem Olamide Olasupo, Qiuju Liang, Chunyi Zhang, Md Shariful Islam, Yansu Li, Xianchang Yu, Chaoxing He

**Affiliations:** 1 Institute of Vegetables and Flowers, Chinese Academy of Agricultural Sciences, Beijing 100081, China; toibraheem@gmail.com (I.O.O.); liyansu@caas.cn (Y.L.); yuxianchang@caas.cn (X.Y.); 2 Biotechnology Research Institute, Chinese Academy of Agricultural Sciences, Beijing 100081, China; liangqiuju@caas.cn (Q.L.); zhangchunyi@caas.cn (C.Z.); sharifulcaas@gmail.com (M.S.I.)

**Keywords:** hidden hunger, folate derivatives, HPLC-MS, residue incorporation, rhizobacteria, greenhouse vegetables, nitrate, photosynthesis

## Abstract

Agronomic biofortification of horticultural crops using plant growth-promoting rhizobacteria (PGPR) under crop residue incorporation systems remains largely underexploited. *Bacillus subtilis* (B1), *Bacillus laterosporus* (B2), or *Bacillus amyloliquefaciens* (B3) was inoculated on soil containing chili residue, while chili residue without PGPR (NP) served as the control. Two hybrid long cayenne peppers, succeeding a leaf mustard crop were used in the intensive cultivation study. Net photosynthesis, leaf stomatal conductance, transpiration rate, photosynthetic water use efficiency, shoot and root biomass, and fruit yield were evaluated. Derivatives of folate, minerals, and nitrate contents in the pepper fruits were also assessed. B1 elicited higher net photosynthesis and photosynthetic water use efficiency, while B2 and B3 had higher transpiration rates than B1 and NP. B1 and B3 resulted in 27–36% increase in pepper fruit yield compared to other treatments, whereas B3 produced 24–27.5% and 21.9–27.2% higher 5-methyltetrahydrofolate and total folate contents, respectively, compared to B1 and NP. However, chili residue without PGPR inoculation improved fruit calcium, magnesium, and potassium contents than the inoculated treatments. ‘Xin Xian La 8 F1’ cultivar had higher yield and plant biomass, fruit potassium, total soluble solids, and total folate contents compared to ‘La Gao F1.’ Agronomic biofortification through the synergy of *Bacillus amyloliquefaciens* and chili residue produced better yield and folate contents with a trade-off in the mineral contents of the greenhouse-grown long cayenne pepper.

## 1. Introduction

In spite of the reduction in caloric malnutrition (acute hunger), micro-nutrient malnutrition, the so-called ‘hidden hunger’, caused by insufficient intake of crucial vitamins and minerals remains a scourge, hitting 2 billion people globally [1], with emphasis on vitamins A, B_1_, B_6_, B_9_, C, and E as well as iron and iodine. Hidden hunger has long term effects on human health, impairing learning ability and productivity, thereby posing a major impediment to socioeconomic development and contributes to the vicious cycle of underdevelopment, which is not limited to the developing world [2]. Tetrahydrofolate (THF) and its derivatives are only synthesized *de novo* by plants and microorganisms; therefore, human solely rely on diets to obtain the amount of folates required for a broad range of their body’s physiological and molecular processes [3]. Rich sources of folates include green leafy vegetables, beans, and certain fruits and fermented foods [4,5,6,7]. Biofortification of crops is a complementary method for alleviating the global burden of micronutrient malnutrition through conventional and traditional plant breeding; metabolic engineering which includes genetic modification; and application of inputs such as fertilizers and bio-stimulants [8], otherwise known as agronomic biofortification. Foliar application of salicylic acid (250 µM, 24 h)—a bio-elicitor—enhanced the total folate content in coriander [9]. García-Salinas et al. [10] reported 24% and 51% enhancement in the folate pools of ripe tomatoes and bananas, respectively, when treated post-harvest with ethylene (100–150 mL/L) compared to the untreated ripened control. Folate deficiency results in severe human disorders, such as infant neural tube defects (NTD), megaloblastic anemia, and aggravates the risks of cardiovascular disease and certain cancers [11,12]. Folic acid supplementation policy in food is in place in many high-income countries, and it has been effective [13,14]. Nonetheless, scientific concern is growing with respect to folic acid supplementation because high folic acid intake poses adverse effects on human health, including risk of prostate, breast and colorectal cancer, and impaired fetal growth and brain development [3,15].

Over the years, efforts are geared towards biotechnologically fortifying major staple foods, for instance, rice, maize, wheat, millet, potatoes, and cassava [3,16], with folate and other vitamins and minerals because they are consumed by most populations of the world for their high calorie contents. Peppers are among the most indispensable spices and culinary herbs, and they are known for their activities as antioxidants, antimicrobials in humans, and a host of nutritional benefits [17,18,19,20]. Kantar et al. [21] studied the folate and vitamins A and C composition of 100 phenotypes of the *Capsicum* spp. and found that some phenotypes actually contained as high folates as other food types that are rich in folate even though processing techniques could affect the stability and ultimate nutritional benefit derivable from folate contents in pepper. Therefore, improving the folate contents of this popular spice by using cost-effective and time saving strategies such as agronomic biofortification could help combat folate deficiency and contribute to nutrition security.

China accounted for 83% of the total global area covered by greenhouse farming, which was approximately 5.6 million in the year 2019 [22]. The typical greenhouses in the country are classified as solar greenhouses and plastic-tunnels with a higher distribution of the former and the latter in the northern and southern parts, respectively [23]. The Chinese solar greenhouse is a peculiar enclosure with plastic roof and north-wall that is dependent on solar energy for plant growth; it produced about 85% of vegetables consumed in the country, creating about 70 million jobs for rural communities in 2014 [24]. The total area of pepper production under greenhouse systems is still soaring in the country [25]. The application of plant growth-promoting rhizobacteria (PGPR) is heightening, with several of these microbials now commercialized in Asia, Europe, and America. Apart from their activities as soil phosphorus and potassium solubilizers and bio-elicitors for plant growth, the use of PGPR in improving yield of crops as biofertilizer and as biocontrol agents for pathogenic diseases has gained extensive research attention [26,27,28,29]. Nonetheless, very little is known about its importance in enhancing the quality of crops as an agronomic biofortification tool.

The use of synthetic fertilizers has resulted in a boom in crop yield and profit for farmers, however, the attendant environmental impact and ultimate health implications, for instance, soil acidification and degradation, water pollution, eutrophication, and dwindling crop yields [30,31,32] are due to the misuse and overdependence on these fertilizers. This necessitates a paradigm shift towards the judicious use of inputs from organic sources as complements or substitutes in farming systems. Proper recycling of organic materials (plants and animal wastes) in soil is a sustainable and environmentally friendly strategy which improves soil physico-chemical and biological properties and crop productivity [33,34]. In situ incorporation of cereal crop residue is practiced over the years to enhance soil quality and increase crop production [35,36,37], but studies on the utilization of horticultural crop residue are limited. There are no available data on the amount of horticultural crop residue generated annually at the national level unlike the cereal crops residue. In spite of this, from the vast production of vegetable crops in China, it can be deduced that the amount of residue from vegetable crops is huge and should be put into efficient use against the practices of burning them in open air or heaping them, which constitute an environmental nuisance. There are indeed fewer reports on the synergistic effects of PGPR and horticultural crop residue on crop–soil interaction, yield, and yield quality. Iqbal et al. [38] reported a yield increase in maize as a result of the incorporation of faba bean residue, single super phosphate and seed inoculation with phosphate solubilizing bacteria. Maize straw and cellulose-decomposing bacteria enhanced soil organic matter and growth of *Malus hupehensis* Rehd [39]. While broccoli residue incorporation increased the abundance of beneficial microbes in the soil fungal community of potato, the inoculation of *Bacillus subtilis* NCD-2 decreased the incidence of verticillium wilt and, consequently, increased crop yield [40].

The current study therefore, sought to unravel the impact of PGPR and chili residue synergy on eight folate derivatives, minerals, and nitrate contents in addition to leaf–gas exchange, plant biomass, and yield of two long cayenne pepper cultivars in a protected cultivation system. This will contribute to the existing pool of knowledge on agronomic biofortification of crops for global food security.

## 2. Materials and Methods

### 2.1. Experimental Site Description

This study was conducted in a solar greenhouse under a mustard-pepper cropping sequence at the Institute of Vegetables and Flowers, Chinese Academy of Agricultural Science (CAAS), Beijing (latitude 39°58′59.7′′ N; 116°18′55.8′′ E), between December 2019 and August 2020. The pre-experiment soil physico-chemical properties and chili residue quality analysis revealed that the soil and residue contained 4.7% and 37.5% total carbon, 0.24% and 3.2% total nitrogen, and 19.1 and 11.7 carbon to nitrogen ratio, respectively, as presented in Table 1.

### 2.2. Experimental Design and Crop Management

The study consisted of four treatments—*Bacillus subtilis* (B1), *Bacillus laterosporus* (B2), *Bacillus amyloliquefaciens* (B3), and NP—which included the sole incorporation of chili residue without PGPR, serving as the control. The PGPR inoculants were obtained from Jining Jinyizhu Biotechnology Co. Ltd., Shandong, China; they were in powdery form, containing 10^10^ cfu/gram. The treatments were arranged in a randomized complete block design with three replications. The chili residue—leaf, stem, and root—was mechanically shredded into 1–3 cm in length and incorporated into the soil to a depth of 20 cm at the rate 5 t ha^−1^ (dry weight) in December 2019. Sunken beds, on which the study was conducted, measured 4.2 m^2^ with 0.7 m inter-bed spacing and were covered with black plastic mulch for solarization effect. The vents of the greenhouse were closed in order to further raise the soil and ambient temperatures for residue decomposition. The average air and soil temperatures rose to 38.5 and 24 °C, respectively, at 34.6–37.7% soil moisture during the solarization period. After four weeks, the greenhouse vents were opened, and the soil and air temperatures declined to 11.8 and 22.0 °C, respectively, while the soil moisture content was 38.0%. Consequently, PGPR inoculants were accordingly worked into the soil (not beyond 20 cm depth) at the rate of 150 kg ha^−1^ and sparingly moistened.

Leaf mustard was grown on these treated plots; subsequently, the land was prepared for the current pepper study, conducted between April and August 2020. This time, there was no new incorporation of chili residue because crop residues, like many other organic materials, are known for not undergoing complete decomposition within such ephemeral period of four months after incorporation. However, one of the reported limitations with PGPR application in cropping systems is their short-term persistence in soil [41]. This was evident by the high throughput sequencing analysis of the soil bacterial community in the rhizosphere after the first crop—leaf mustard (Unpublished). Therefore, a repeat of the PGPR inoculation was carried out before the long cayenne pepper seedlings were planted in the current study, following the exact procedure and rate of application as earlier reported in Section 2.2.

Two high yielding hybrid cultivars of long cayenne pepper namely—‘La gao F1’ and ‘Xin Xian La 8 F1’—were purchased from Zhongdu Hi-tech Seed Co. Ltd., Sichuan, China and Yashuyuan Seedling Co. Ltd., Guangzhou, China, respectively. The seedlings were transplanted at four weeks after sowing, which corresponded to 4–6 true leaf stage, at a spacing of 60 cm × 30 cm, inter and intra row under drip irrigation. There was a blanket application of one-third of the recommended fertilizer application rate for capsicum production in order to make mineral nutrients readily available for the activation of the inoculated and native microbes for their numerous physiological processes and to facilitate the decomposition of the incorporated residue [42]. The applied rate of chemical fertilizer translated to 84.7 kg ha^−1^ N, 25.7 kg ha^−1^ P_2_O_5_, and 116.7 kg ha^−1^ K_2_O [43].

### 2.3. Agronomic Parameters

Leaf–gas exchange parameters: net photosynthesis, stomatal conductance, intercellular CO_2_ and transpiration rate, and photosynthetic water use efficiency were determined by CIRAS-3 portable photosynthesis system (Massachusetts, USA) between 10 a.m. and noon. Long cayenne pepper fruits were harvested weekly between 12 and 15 weeks after transplanting (WAT) when they were red ripe but still fresh. Samples of fruits were rinsed, ground homogenously, freeze dried, and stored under −80 °C for mineral and nitrate analysis. Fruit yield and yield morphological attributes such as fruit length and width and the number of fruits were recorded. Shoot dry weight, root dry weight, and plant cumulative dry biomass were determined at 15 WAT.

### 2.4. Determination of Mineral and Nitrate Contents in Long Cayenne Pepper Fruits

Calcium, magnesium, and potassium were determined according to the procedure of [44]. An amount of 350 mg of ground and homogenized sample was placed in a digestion tube, and 5 mL of HNO_3_ was pipetted into the tubes. The tubes were swirled gently during nitric acid pre-digestion at 175 °C until the solution began to steam and was cooled for 30 min. Thereafter, 4 mL of 30% hydrogen peroxide was added to each tube, followed by another round of gentle swirling, then the tubes were returned to the digestion block and set at the previous temperature. As soon as reaction started, the tubes were removed from the block and placed in the cooling rack for the reaction to continue; 2 mL concentrated nitric acid was added and heating continued. The sequences of heating, tube cooling, and acid addition were carried out every 15 min until all tubes were completely digested and were finally cooled at room temperature. Ash samples were diluted to 10 mL with distilled water and filtered using Whatman ashless #540 filter paper, and the determination of the mineral elements was conducted by inductively coupled plasma-optical emission spectroscopy (ICP-OES). Extraction of nitrate from the pepper samples was performed according to [45]. Briefly, 1 g sample was transferred into 125 mL conical flask, about 100 mL hot water (70–80 °C) was added, and the mixture was heated for 15 min in a boiling water bath. After cooling, the content was transferred to a volumetric flask and diluted to 200 mL with ultra-pure water. Sample extracts were further analyzed by ion chromatography for nitrate content.

### 2.5. Determination of Folate in Long Cayenne Pepper Fruits

The following standards and reagents were used: 10-formyl-folic acid (10-F-FA), 5, 10-methenyl-tetrahydrofolate (5,10-CH=THF), 5-formyl-tetrahydrofolate (5-F-THF), 5-methyl-tetrahydrofolate (5-M-THF), dihydrofolate (DHF), folic acid (FA), tetrahydrofolate (THF), and methotrexate (MTX) standards were purchased from Shircks Laboratories. The purity of the folate standards was >95%. Sodium phosphate monobasic (NaH_2_PO_4_), sodium phosphate dibasic (Na_2_HPO_4_), sodium ascorbate, β-mercaptoethanol, and α-amylase (from *Aspergillus oryzae*, ~30 units/mg) were purchased from Sigma-Aldrich. Ultra-pure water was purified on a Heal Force ultra-pure water system. Acetonitrile and formic acid (LC-MS grade) were purchased from Fischer Scientific. Rat serum was purchased from Solarbio Life Sciences and was aliquoted into 1 mL portions in 1.5 mL tubes upon arrival and subsequently stored in a −80 °C freezer before use. The α-amylase was freshly prepared in water with the concentration of 40 mg/mL and utilized the same day. The endogenous folates in rat serum were expunged by incubation with one-tenth (*w*/*w*) of activated charcoal for 1 hr on ice, followed by centrifugation at 15,000 rpm and 4 °C for 30 min (Sigma 3K15), and the supernatant was used for the folate extraction.

Freshly harvested, fully ripe pepper fruits were macerated and homogenized using mechanical kitchen blender, about 2 g was used for the determination of moisture content, and the rest was set aside for folate determination following the description of [6]. The extraction buffer (50 mM phosphate buffer, pH 7.0; 0.5% (*w*/*v*) sodium ascorbate; 0.2% β-mercaptoethanol) was freshly prepared. MTX at a final concentration of 20 ng/mL was used as the internal standard and added to the extraction buffer at the commencement of the extraction process. Furthermore, 1 mL of the extraction buffer was added to 50 mg of the pepper paste and mixed. The mixture was immediately boiled for 10 min in a water bath and cooled on ice; then, 30 µL of rat serum was added, and the resultant content was incubated at 37 °C for 4 h to convert polyglutamate folates into monoglutamates. The samples were then boiled for 10 min to inactivate rat conjugase, cooled on ice for 10 min, centrifuged at 13,000 rpm at 4 °C for 10 min, and the supernatants were transferred into 3 kDa ultra-filtration tubes (Millipore) and centrifuged at 13,000 rpm at 4 °C for 20 min. The obtained solution was directly used for folate determination by high performance liquid chromatography with tandem mass spectrometry (HPLC-MS/MS).

Following the descriptions of [46] and further elucidations by [6], separation of folate into its derivatives (5-methyltetrahydrofolate (5-MTHF); 5–formyltetrahydrofolate (5-F-THF); 10-formylfolic acid (10-F-FA); tetrahydrofolate (THF); folic acid (FA); Dihydrofolic acid (DHF); 5, 10-Methylenetetrahydrofolate (5-10-CHTHF); and MeFox (pyrazino-*s*-triazine derivative of 4α-hydroxy-5-methyltetrahydrofolate) and their quantifications were carried out by using chromatographic separation performed in an Agilent 1260 HPLC unit. System operation, data acquisition, and data analysis were performed by using the Agilent Mass Hunter software. In calculating total folate contents reported in this study, MeFox was excluded as found in [10,40] because MeFox, an oxidative product of 5-methyltetrahydrofolate [47], is not known to be biologically active; therefore, it does not contribute to the amount of total folates [48].

### 2.6. Determination of Moisture Content

Moisture contents were determined by drying 2 g (initial weight) of samples in a vacuum oven at 70 °C overnight and then weighed. The moisture content was calculated as the difference between the two weights.

### 2.7. Statistical Analysis

Data were subjected to analysis of variance, and their means were separated by Tukey’s significant test at 5% probability level using R statistical package [49]. Correlograms based on the Pearson’s correlation of agronomic and quality parameters of each pepper variety was conducted with the corrplot library [50], also on the R software.

## 3. Results and Discussions

### 3.1. Leaf–Gas Exchange of Cayenne Pepper

Inoculating soil containing chili residue with PGPR influenced (*p* < 0.05) the leaf–gas exchange of the plant (Table 2). Residue with *B. subtilis* (B1) elicited a net photosynthesis of 26.2 µmolm^−2^ s^−1^ CO_2_, followed by B2 and B3, which had 25.1 and 25.3 µmolm^−2^ s^−1^ CO_2_, respectively, while NP had the least (20.8 µmolm^−2^ s^−1^ CO_2_). The transpiration rates of pepper plants in B2 and B3 treatments were significantly higher than those in B1 and NP plots by 19–22%. Photosynthetic water use efficiency was higher (*p* < 0.05) in B1 plants than other PGPR treatments by 14–15%, but it was 19.5% greater than NP (Table 2). Leaf stomatal conductance across the treatments were at par. However, the two cultivars of cayenne pepper responded similarly in terms of the leaf–gas exchange parameters evaluated (Table 2).

That inoculation of rhizobacteria improved net photosynthesis, transpiration rate, and photosynthetic water use efficiency (Table 2), corroborating earlier reports on leaf–gas exchange enhancements which are modulated by plant hormones induced by PGPR. Phyto-hormone synthesis is among the direct mechanisms of plant growth promotion by certain rhizobacteria [51,52,53]. These hormones and their interactions with secondary messengers are crucial endogenous factors regulating stomatal movement and, thus, transpiration in plants [54]. Auxins, most notably Indole-3-acetic acid (IAA), are powerful molecules that are naturally produced by plants and also induced by PGPR. They are involved in almost every aspect of plant physiology, controlling cell division, expansion, and differentiation [55,56], which have direct impacts on overall plant photosynthesis. It is, however, worthy of note that while B1 improved net photosynthesis, B2 and B3 rather enhanced transpiration rates in the current study.

### 3.2. Yield Attributes and Plant Biomass of Cayenne Pepper Cultivars

As presented in Table 3, inoculating soil containing chili residue with PGPR enhanced (*p* < 0.05) fruit yield of cayenne pepper cultivars. B1 and B3 had 27–36% superior yield compared to B2 and NP. Furthermore, V43 produced 22.4% higher fruit yield than V6. As obtained in yields, similar effects of cultivar and PGPR were recorded on fruit count whereby B1 produced 27% more fruits than B2 and NP, while B3 produced 36% and 57% more fruits than B2 and NP, respectively, even though the numbers of fruits produced by plants on B1 and B3 plots were at par (Table 3). V43 was found with longer fruits (41 cm) than V6 (33 cm), although PGPR did not affect fruit numbers. In terms of plant dry biomass, the shoot and plant dry biomass were markedly increased by B1 and B2 compared to B3 and NP. V43 also had an increased shoot and plant dry biomass than V6. Both cultivar and PGPR did not influence root biomass (Table 3).

Numerous studies have been conducted to investigate the effects of either crop residue or PGPR on crop performance, as earlier cited in Section 1. However, there is an extreme paucity of reports on the interactive effects of both crop residue and PGPR on horticultural and staple food crops. Therefore, the current study is among the first to document the synergistic impacts of vegetable crop residue and rhizobacteria inoculation on the performance of vegetable crops. The yield increase recorded in B1 and B3 relative to B2 and NP (Table 3) conforms with previous studies. *Bacillus amyloliquefaciens plantarum* significantly improved the yield of *Brassica oleracea* var. *acephala* compared to other species of PGPR in a greenhouse system [57]. When seed potato inoculated with *Bacillus subtilis* NCD-2 was planted on a soil on which broccoli residue was incorporated, the yield of potato increased by 13% and 8% compared to sole *B. subtilis* and sole broccoli residue treatments, respectively [40]. Wei et al. [34] reported yield increases in cucumber and tomato as a result of vegetable crop residue incorporation in a solar greenhouse under a crop rotation system. As recorded in the present study (Table 3), the sole inoculation of PGPR increased the plant dry biomass of tomato [58]. Such an increase in yield of cayenne pepper in the current study in response to the inoculation of *B. subtilis* or *B. amyloliquefaciens* on soil containing chili residue indicates a desirable benefit for farmers at a relatively low cost, and it contributes to the existing knowledge on sustainable vegetable production. Essentially, any biofortification strategy could only be successful and sustainable if it delivers a high and profitable yields for farmers because this will motivate them to invest their resources in producing such biofortified crops to nourish the populace.

It would be interesting however, to investigate how much of the conventional synthetic fertilizer this nexus of residue and PGPR can substitute in the nutrition of pepper crop in order to provide a stronger basis for discouraging farmers’ overdependence on synthetic fertilizers. Furthermore, this study establishes the higher yielding capacity of ‘V43’ over ‘V6’ with a difference of 22.4%, despite the two of them had similar yield magnitudes when grown in a conventional system without PGPR and crop residue incorporation in a previous trial (Unpublished).

### 3.3. Quality Attributes of Cayenne Pepper Fruits

#### 3.3.1. Total Soluble Solids, Mineral Contents, and Nitrate Accumulation

The pepper cultivars exhibited varying (*p* < 0.05) contents of total soluble solids and mineral elements but had comparable nitrate load (Table 4). V43 cultivar contained a higher brix value (4.6%) than V6 (4.0%). Total soluble solids were not affected by the treatments. Inoculating soil containing chili residue with *B. subtilis* resulted in a drastic reduction (*p* < 0.05) in nitrate content in the cayenne pepper fruits. This was 41%, 30.6%, and 8.6% lower than the nitrate loads in fruits harvested from B2, B3, and NP plots, respectively. Nitrate content was, however, not influenced by cultivar (Table 4).

The lower amount of nitrate in pepper fruits harvested from B1 plot (Table 4) suggests the ability of this inoculant and other native organisms to effectively immobilize NO_3_^−^ in the soil, without necessarily affecting the optimum supply of NO_3_^−^ N back to the soil during nitrification for the plant’s uptake. Such nitrate, if not immobilized by the organisms, could have hitherto become excessive and unutilized by the plant and, consequently, be distributed from the root through the xylem tissues to all parts of the shoot system including the fruit. During the storage and processing of plant produce, excess NO_3_^−^ that remains unassimilated in plant tissues can be enzymatically converted to NO_2_^−^ [59]. The NO_3_^−^ otherwise ingested by humans can be reduced to NO_2_^−^ through the activities of gut microorganisms [59]. The resultant NO_2_^−^ is a strong carcinogen and results in many diseases including fetal birth defects and methemoglobinemia in children [60]. On the other hand, the process of nitrification seemed to be rather more rapid due to the inoculation of B2 and B3; this could be responsible for the higher nitrates found in the pepper fruits harvested in those treatments (Table 4).

Sole residue without PGPR inoculation enriched (*p* < 0.05) cayenne pepper fruits in calcium and potassium by 8–16% and 6–20%, respectively, than the inoculated treatments. This runs parallel to some previous reports that did not involve crop residue incorporation. *B. amyloliquefaciens plantarum* enhanced nitrogen, potassium, and calcium contents of a collard plant than other inoculants [57]. Foliar N and K were markedly increased due to the inoculation of potato tubers with *Achromobacter xylosoxidans* [61]. Fruit magnesium content in B2 and NP plants were 6–21% significantly higher than those from B1 and B3 plants (Table 4). The nutritional enhancement of cayenne pepper recorded in uninoculated plots in the present study indicates that residue incorporation could have outweighed the effect of PGPR inoculated because it was expected that PGPR would increase the mineral composition of the pepper fruit. Calcium and magnesium contents of tomato were increased as a result of faba bean residue incorporation, without PGPR inoculation [62]. Cultivar effect was only observed in calcium and potassium (Table 4). V43 contained higher calcium, while V6 had higher potassium content in the fruit.

#### 3.3.2. Folate Derivatives

There were significant effects of residue and PGPR synergy and cultivar on folate derivatives in the cayenne fruits (Table 5). The inoculation of soil containing chili residue with each of *B. laterosporus* and *B. amyloliquefaciens* significantly increased 5-MTHF content by 21.8–25.1% and 24–27.5%, respectively, compared to other treatments. B2 elicited 28.8–48.6%, 26–52.2% higher THF, and 5,10-CHTHF contents than other treatments, respectively. Furthermore, B2 enhanced 5-F-THF content than B1, B3, and NP by 31.8%, 23.5%, and 43.8%, respectively. For MeFox content in the cayenne fruits, the same B2 increased this derivative by 23%, 3.7%, and 14.4% than B1, B3, and NP, respectively. Excluding MeFox, the total folate content in B2 and B3—grown cayenne pepper was 11.9 and 11.4 µg/100 g, respectively, followed (*p* < 0.01) by B1 (8.9 µg/100 g) and NP (8.3 µg/100 g). Further, V43 cultivar contained higher amounts of THF, 5-F-THF, MeFox, and ultimately total folate compared to V6, which conversely contained higher 5,10-CHTHF, DHF, and FA (Table 5).

Of specific interest is the remarkable increase in 5-MTHF derivative of folate in the cayenne pepper as a result of B2 and B3 treatments. Strobbe and Straeten [63] submitted that in biofortification programs, the most desirable folate vitaminer is 5-MTHF, and this is due to its stability and bio-activity. In the current study, 5-MTHF accounted for most of the bio-active derivatives of the total folate recovered from the cayenne pepper regardless of the cultivar. Nonetheless, there are very few findings available on folate fortification by agronomic techniques. That inoculation of B2 and B3 elicited a marked increase in total folate of pepper in the current study is consistent with the report of [64] that PGPR inoculation remarkably improved folate content in moringa leaves. The authors discovered an upregulation of the folate biochemical pathway gene, dihydrofolate reductase thymidylate synthase (DHFR-TS), as a result of PGPR application on moringa plants. Agronomic biofortification is a fast and affordable method for increasing nutrients in food crops [65], and it can complement other biofortification efforts such as conventional and traditional breeding, and genetic modification. Generally, the application of PGPR is considered a new and attractive method of biofortifying crops; at the same time, it offers the advantage of reducing the use of chemical fertilizers and pesticides [65]. However, the applicability of PGPR in fortifying food crops with vitamins and minerals constituting hidden hunger and the mechanisms underlying these biofortifications need to be more widely explored.

It is noteworthy that the inoculation of *B. amyloliquefaciens* on soil containing chili residue in the present study, which elicited a superior yield of cayenne fruit compared to other inoculants, also enhanced the folate content in the fruits but had a trade-off in terms of mineral contents—calcium, magnesium, and potassium. Moreover, going by the recommended daily allowance (RDA) for folate intake, which is 200 µg for adult males, 180 µg for adult females, and 400 µg for pregnant women [66], it can be inferred that folate enhancement in pepper by PGPR and chili residue nexus in the current study is relatively lower compared to the RDA values. Even though MeFox is biologically inactive in microbiological assays [67], large amounts of this folate derivative were previously found in seeds of pulse crops [68], and in different kinds of food and vegetables including cauliflowers, carrots, and peas [46]. Little is known about the mechanisms underlying the accumulation of MeFox in the current pepper study (ranging between 24.3 and 30.4 µg/100 g), which was found to be far more than the contents of other folate derivatives analyzed.

### 3.4. Correlations between Folate Derivatives and Mineral Contents of Cayenne Pepper

**Figure 1:**
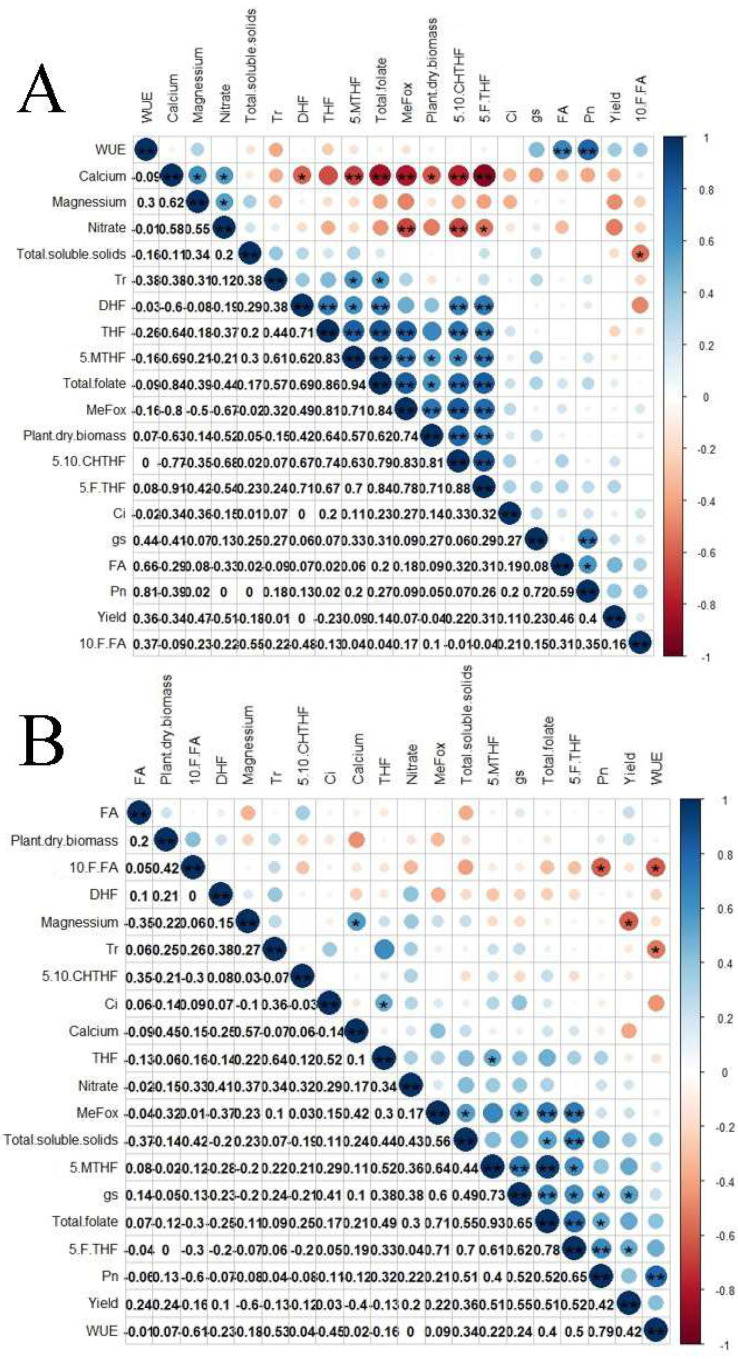
Correlogram based on Pearson’s correlation analysis between the folate derivatives and mineral contents of fruits of (**A**) ‘Xin Xian La 8 F1’cultivar—V43 and (**B**) ‘La Gao F1’ cultivar—V6. Correlation coefficients on the lower and left side of the correlogram; ‘*’ and ’**’ indicate significance of *p* value at 5% and 1% probability levels, respectively. Tr: transpiration rate; Ci: intercellular CO_2_; gs: stomatal conductance; Pn: net photosynthesis; WUE: photosynthetic water use efficiency; 5.MTHF: 5-methyltetrahydrofolate; 5.F.THF: 5-formyltetrahydrofolate; 10.F.FA: 10-formylfolic acid; THF: tetrahydrofolate; FA: folic acid; DHF: dihydrofolic acid; 5.10.CHTHF: 5, 10—Methylenetetrahydrofolate; MeFox: pyrazino—*s*—triazine derivative of 4α—hydroxy—5—methyltetrahydrofolate.

In ‘Xin Xian La 8 F1’ cultivar (V43), fruit calcium content correlated significantly with potassium (0.73), magnesium (0.62), and nitrate (0.52), as presented in Figure 1A. A significant correlation existed between fruit potassium and magnesium (0.87). The total folate content correlated significantly with other derivatives such as DHF (0.68); THF (0.86); 5-MTHF (0.88); 5-F-THF (0.88); 5, 10-CHTHF (0.82); and MeFox (0.86), as shown in Figure 1A. However, notable inverse correlations were observed between mineral contents and folate derivatives. Calcium correlated negatively with DHF (−0.58); THF (−0.64); 5-MTHF (−0.69); 5, F-THF (−0.88); 5, 10-CHTHF (−0.82); MeFox (−0.78); and total folate (−0.82). With the exceptions of FA, DHF, and THF, potassium correlated negatively and significantly with 5-MTHF (−0.52), 5-F-THF (−0.60), 5,10-CHTHF (−0.56), MeFox (−0.70), and total folate (−0.62) (Figure 1A). These results are largely consistent with the report of [6] that 5-MTHF was not only the major contributor to the total folate content in major foods but also correlated highly (0.96) with total folate. Certain agronomic parameters also exhibited relationships with quality indices (Figure 1A). Significant correlations existed between the following: Tr and total folate (0.57), Pn and WUE (0.81) but neither yield, and Ci nor g_s_ correlated with total folate content.

Unlike the V43 cultivar, fewer correlations were recorded between the mineral contents and folate derivatives in ‘La Gao F1’ cultivar (V6), as presented in Figure 1B. Calcium correlated significantly with magnesium (0.56) and potassium (0.55). A positive and significant correlation was also observed between magnesium and potassium (0.80). On the account of folate derivatives, total folate correlated significantly with THF (0.62); 5, F-THF (0.63); 5-MTHF (0.90); and MeFox (0.71), as shown in Figure 1B. It is, however, noteworthy that mineral contents in fruits had no significant correlation with folate derivatives in this cultivar of cayenne pepper. In terms of agronomic indices (Figure 1B), significant correlations occurred between the following: g_s_ and total folate (0.65); Pn and total folate (0.52). Furthermore, yield had a positive correlation with g_s_ (0.55) but a negative correlation with Mg content (−0.60).

## 4. Conclusions

Agronomic biofortification through the synergy of *Bacillus amyloliquefaciens* and chili residue produced better yields and folate contents but with a trade-off in the mineral contents of long cayenne peppers. Meanwhile, the ‘Xin Xian La 8 F1’ cultivar is superior in terms of yield and plant biomass, fruit potassium, total soluble solids, and total folate contents compared to ‘La Gao F1.’

## Figures and Tables

**Table 1:** Soil physico-chemical properties and chili residue quality.

Sample	pH	EC (µS cm^−1^)	OM (%)	TC (%)	TN (%)	C:N	TP (g kg^−1^)	TK (g kg^−1^)	Ca (mg kg^−1^)	Mg (mg kg^−1^)	Sand (%)	Silt (%)	Clay (%)
Soil	8.0	394	4.3	4.7	0.24	19.1	2.1	17.4	186.4	31	35	40	25
Residue	NA	NA	NA	37.5	3.2	11.7	12	0.28	100.7	42.4	NA	NA	NA

EC: electrical conductivity; OM: organic matter; TC: total carbon; TN: total nitrogen; TP; total phosphorus, TK: total potassium; NA; not analyzed; (*n* = 3).

**Table 2:** Leaf–gas exchange parameters of tunnel-grown cayenne pepper cultivars in response to PGPR and chili residue synergy.

Cultivar	Pn (µmol m^−2^ s^−1^ CO_2_)	gs (mmol m^−2^ s^−1^ H_2_O)	Tr (mmol m^−2^ s^−1^ H_2_O)	WUE (µmolCO_2_/mmolH_2_O)
V43	23.65a	2.067a	12.03a	1.992a
V6	24.12a	1.843a	11.63a	2.088a
*p* ≤ 0.05	ns	ns	ns	ns
Treatment				
B1	26.24a	1.928a	11.09b	2.382a
B2	25.08ab	2.124a	12.63a	2.038ab
B3	25.28ab	2.371a	12.61a	2.02ab
NP	20.82c	1.475a	10.89b	1.917b
*p* ≤ 0.05	**	ns	*	*

V43: ‘Xin Xian La 8 F1’ cultivar; V6: ‘La Gao F1’ cultivar; B1: Residue + *Bacillus subtilis;* B2: Residue + *Bacillus laterosporus;* B3: Residue + *Bacillus amyloliquefaciens;* NP: Sole residue; Pn: Net photosynthesis rate; gs: Leaf stomatal conductance; Tr: Transpiration rate. Means with the same letter in a column are not significantly different according to Tukey’s test at 1% (**) or 5% (*) probability levels, respectively; ns: no significant difference between means (*n* = 3).

**Table 3:** Yield and biomass of cayenne pepper cultivars in response to PGPR and chili residue.

Cultivar	Fruit Length (cm)	Number of Fruits (ha^−1^)	Shoot Dry Weight (gplant^−1^)	Root Dry Weight (gplant^−1^)	Plant Dry Weight (gplant^−1^)	Fruit Yield (tha^−1^)
V43	41.08a	651,066a	59.9a	9.694a	69.61a	22.79a
V6	33.68b	505,125b	43.6b	9.982a	53.63b	17.68b
*p* ≤ 0.05	**	**	**	ns	**	**
Treatment						
B1	37.87a	674,376a	58.5a	9.31a	67.80a	23.60a
B2	36.75a	489,116b	56.9a	11.14a	68.06a	17.12b
B3	37.20a	771,655a	54.9ab	9.73a	64.58ab	27.01a
NP	37.56a	490,023b	51.9ab	10.26a	62.18ab	17.15b
*p* ≤ 0.05	ns	**	*	ns	*	**

V43: ‘Xin Xian La 8 F1’ cultivar; V6: ‘La Gao F1’ cultivar; B1: Residue + *Bacillus subtilis;* B2: Residue + *Bacillus laterosporus;* B3: Residue + *Bacillus amyloliquefaciens;* NP: Sole residue; means with the same letter in a column are not significantly different according to Tukey’s test at 1% (**) or 5% (*) probability levels, respectively; ns: no significant difference between means (*n* = 3).

**Table 4:** Fruit mineral and nitrate contents of tunnel-grown cayenne pepper cultivars in response to PGPR and chili residue interaction.

Cultivar	Calcium (mg kg^−1^)	Magnesium (mg kg^−1^)	Potassium (mg kg^−1^)	TSS (%)	Nitrate (mg kg^−1^)
V43	106.75b	144.13a	2370.3a	4.595a	266.0a
V6	116.21a	147.37a	2191.4b	4.022b	250.3a
*p* ≤ 0.05	**	ns	*	**	ns
Treatment					
B1	100.78b	138.00bc	2144.2bc	4.238a	204.2c
B2	108.50b	155.25a	2288.3ab	4.710a	288.3a
B3	98.97b	122.00c	1950.0c	4.228a	266.7ab
NP	118.67a	146.83a	2441.0a	4.005a	221.7bc
*p* ≤ 0.05	**	**	**	ns	**

V43: ‘Xin Xian La 8 F1’ cultivar; V6: ‘La Gao F1’ cultivar; B1: Residue + *Bacillus subtilis;* B2: Residue + *Bacillus laterosporus;* B3: Residue + *Bacillus amyloliquefaciens;* NP: Sole residue; TSS: Total soluble solids; means with the same letter in a column are not significantly different according to Tukey’s test at 1% (**) or 5% (*) probability levels, respectively; ns: no significant difference between means (*n* = 3).

**Table 5:** Folate derivatives of cayenne pepper fruits as influenced by PGPR and chili residue synergy.

Cultivar	Moisture (%)	THF (µg/100 g)	5-MTHF (µg/100 g)	5,10-CHTHF (µg/100 g)	10-F-FA (µg/100 g)	5-F-THF (µg/100 g)	DHF (µg/100 g)	FA (µg/100 g)	MeFox (µg/100 g)	Total Folate α (µg/100 g)
V43	94.4	0.400a	7.057a	0.574b	0.282a	2.166a	0.069b	0.002b	32.23a	10.55a
V6	94.1	0.315b	6.686a	0.795a	0.258a	1.379b	0.105a	0.024a	25.23b	9.56b
*p* ≤ 0.05		**	ns	**	ns	**	*	*	**	*
Treatment										
B1	94.6	0.304b	6.077b	0.636b	0.248a	1.600bc	0.056a	0.024a	24.31c	8.94b
B2	93.9	0.504a	7.771a	1.048a	0.15a	2.345a	0.113a	0.004a	31.59a	11.94a
B3	93.4	0.359b	8.026a	0.775ab	0.316a	1.794b	0.137a	0.015a	30.42ab	11.42a
NP	95	0.259b	5.817b	0.501b	0.35a	1.317c	0.056a	0.006a	27.03abc	8.31b
*p* ≤ 0.05		**	**	**	ns	**	ns	ns	*	**

V43: ‘Xin Xian La 8 F1’; V6: ‘La Gao F1’; B1: *Bacillus subtilis*; B2: *Bacillus laterosporus*; B3: *Bacillus amyloliquefaciens*; NP: Sole residue without PGPR. α^:^ Excluding MeFox. Means with the same letter in a column are not significantly different according to Tukey’s test at 1% (**) or 5% (*) probability levels, respectively; ns: no significant difference between means (*n* = 3).

## Data Availability

Data are available with the corresponding author upon reasonable request.
